# Nanoparticle-Based Drug Delivery Systems for Induction of Tolerance and Treatment of Autoimmune Diseases

**DOI:** 10.3389/fbioe.2022.889291

**Published:** 2022-04-06

**Authors:** He Li, Yong-Guang Yang, Tianmeng Sun

**Affiliations:** ^1^ Key Laboratory of Organ Regeneration and Transplantation of Ministry of Education, Institute of Immunology, The First Hospital, Jilin University, Changchun, China; ^2^ National-local Joint Engineering Laboratory of Animal Models for Human Diseases, Changchun, China; ^3^ Department of Rehabilitation Medicine, The First Hospital, Jilin University, Changchun, China; ^4^ International Center of Future Science, Jilin University, Changchun, China

**Keywords:** Antigen-specific tolerance, autoimmune diseases, nanoparticle, tolerogenic DC, drug delivery system

## Abstract

Autoimmune disease is a chronic inflammatory disease caused by disorders of immune regulation. Antigen-specific immunotherapy has the potential to inhibit the autoreactivity of inflammatory T cells and induce antigen-specific immune suppression without impairing normal immune function, offering an ideal strategy for autoimmune disease treatment. Tolerogenic dendritic cells (Tol DCs) with immunoregulatory functions play important roles in inducing immune tolerance. However, the effective generation of tolerogenic DCs *in vivo* remains a great challenge. The application of nanoparticle-based drug delivery systems in autoimmune disease treatment can increase the efficiency of inducing antigen-specific tolerance *in vivo*. In this review, we discuss multiple nanoparticles, with a focus on their potential in treatment of autoimmune diseases. We also discuss how the physical properties of nanoparticles influence their therapeutic efficacy.

## 1 Introduction

Autoimmune diseases result from genetic factors, viral or bacterial infections, and other causes such as abnormal activation of immune cells in the body, which result in the immune destruction of host tissues or organs. According to statistics published online by the American Autoimmune Diseases Association, more than 100 autoimmune diseases affect approximately 24 million people in America (80% are women). Furthermore, approximately 5–10% of the U.S. population has one or more autoimmune diseases. Abnormal activation of T lymphocytes and autoantibodies are often detected in patients, affecting particular organs. These diseases include Hashimoto’s thyroiditis (thyroid gland), pernicious anemia (stomach), Addison’s disease (adrenal glands), and type 1 diabetes (pancreas). These diseases can also involve multiple organs and tissues, such as rheumatoid arthritis, systemic lupus erythematosus (SLE), and dermatomyositis. Autoimmune disease is often repeated with chronic delay. Most patients often display tissue destruction and residual complications during clinical diagnosis. The current treatment for autoimmune diseases involves the administration of broad-spectrum, nonspecific, anti-inflammatory, or immunosuppressive drugs (such as cyclosporine, tacrolimus, or corticosteroids). These treatments mainly reduce the proliferation of inflammatory cells and inhibit the immune reactions in the body, which can alleviate clinical symptoms but cannot fundamentally cure the disease and eliminate complications. Moreover, long-term and extensive use of immunosuppressants and cytotoxic drugs will reduce the body’s normal immune response and increase the potential risk for developing cancer and infections (Chandrashekara, 2012; https://autoimmune.org/resource-center/about-autoimmunity/; Wikins, 2012; Yu et al., 2018; Angum et al., 2020). In recent decades, efforts have been made to focus on developing therapies that can specifically suppress immunity without impairing normal immune function, with the ultimate goal of restoring immune homeostasis (Yang and Santamaria, 2021).

**FIGURE 1:**
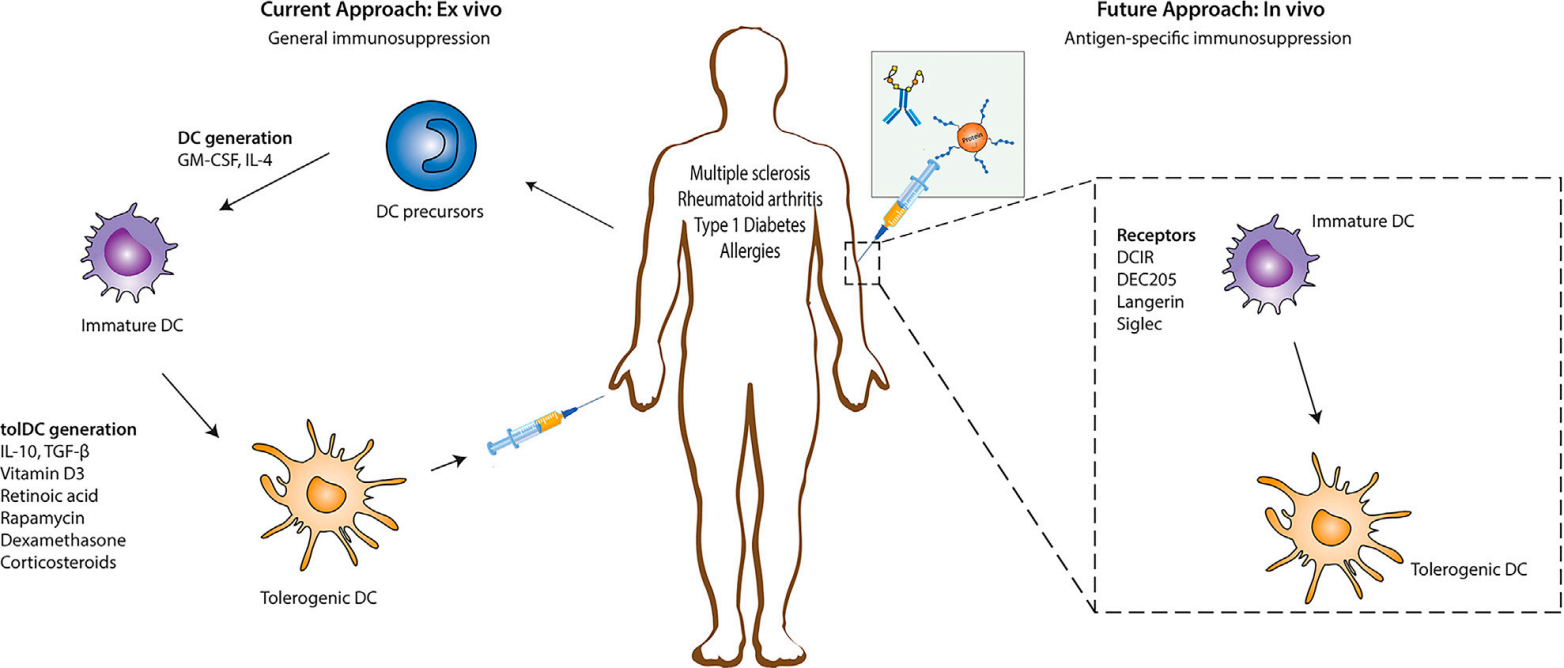
Immune regulation strategy based on DC. Reprogramming DC to induce a tolerogenic phenotype to treat autoimmune diseases. Tolerogenic DC is mainly induced *in vitro*. First, DC precursor cells were isolated from bone marrow and stimulated to differentiate into immature DC (imDC). Then, immunomodulatory agents (such as vitamin D3, rapamycin, retinoic acid, and specific cytokines) were added to induce the differentiation of imDC into tolerogenic DC. Subsequently, reinjecting into patients to suppress inflammation. Another strategy for inducing tolerogenic DC is directly targeting receptors on the DC surface (such as DEC205, langerin, and Siglec receptors). Reproduced with permission from (Castenmiller et al., 2021).

Compared with other immunosuppressive treatments, tolerogenic dendritic cells (Tol DCs) with immunoregulatory functions have attracted much attention to treat autoimmune diseases as they play important roles in inducing and maintaining immune tolerance (Castenmiller et al., 2021). Currently, it is expensive to obtain autologous tolerogenic DC *in vitro,* and there is a possibility of failure *in vivo* after transfusion (Lehmann et al., 2016). Furthermore, *in vitro* tolerogenic DCs provide nonspecific immunosuppression. However, generating tolerogenic DCs to target-specific autoimmune cells requires loading these DCs with disease-specific autoantigens (Stabler et al., 2019; Cifuentes-Rius et al., 2021). Antigen-specific immunotherapy is ideal for treating autoimmune diseases and allergies and preventing allograft rejection (especially executing the modification *in situ*). The advantage of antigen-specific immunotherapy is the inhibition of autoreactive inflammatory T cells and induction of antigen-specific immune suppression without impairing normal immune function. A feasibility strategy *in vivo* focuses on recognizing and internalizing antigens through surface receptors of DCs such as DEC205 and C-type lectin receptors family (macrophage galactose type C-type lectin and MGL), dendritic cell-specific intercellular adhesion molecule-3-grabbing nonintegrin (DC-SIGN), and mannose receptor (MR) (Zizzari et al., 2015; Castenmiller et al., 2021) which lead to immune tolerance. These receptors can trigger different signaling pathways that affect APC functions and determine the polarization of T cells (Geijtenbeek and Gringhuis, 2009). While the activation status of the DCs controls the induction of tolerogenic DCs with receptors, the acquired tolerogenic effect disappears in the presence of pro-inflammatory modulators (Hawiger et al., 2004; Takenaka and Quintana, 2017) and other immune cells expressing similar receptors as those being targeted (Castenmiller et al., 2021). Another reprogramming DC approach is based on nanoparticle administration *in vivo*. The nanoparticle is a new carrier system designed to target the innate immune cells at a specific size, charge, and chemical modification as required, significantly improving drug loading capacity and bioavailability. In particular, the natural affinity of phagocytes for nanoparticles makes them a powerful tool for initiating and modulating immune responses (Kishimoto and Maldonado, 2018). The intervention of autoimmune response based on nanoparticles is mainly focused on following two aspects: 1) tolerance is induced by targeting antigen-presenting cells (Liu et al., 2019) and 2) tolerance is induced by directly targeting autoreactive lymphocytes (Feldmann and Steinman, 2005; Umeshappa et al., 2019; Umeshappa et al., 2020). The ultimate goal of all these methods is to induce tolerance through various mechanisms, including autoreactive T cell anergy, apoptosis, and the induction of Tregs or Bregs that can be used in tolerant immunotherapies (Figure 1) (Prosperi et al., 2017). Antigen-specific immunotherapy based on nano-delivery strategies targeted auto-reacting lymphocytes and antigen-presenting cells such as macrophages (Montes-Cobos et al., 2017), dendritic cells (Lewis et al., 2019), B cells (Stensland et al., 2021), monocytes (Casella et al., 2020), and neutrophils (Sherr et al., 2008). They co-loaded a specific amount of pathogenic antigen through covalent binding, biological coupling, and electrostatic adsorption or co-delivered some immunomodulatory substances that contributed to tolerance simultaneously, thus performing DC reprogramming *in situ*. This article reviews the recent progress of nanotechnology in inducing antigen-specific tolerance *in vivo*. Here, we examine how properties of nanoparticles affect immune tolerance and common strategies for nanoparticles to induce immune tolerance.

## 2 Antigen-Presenting Cells Play an Essential Role in Immune Tolerance

**FIGURE 2:**
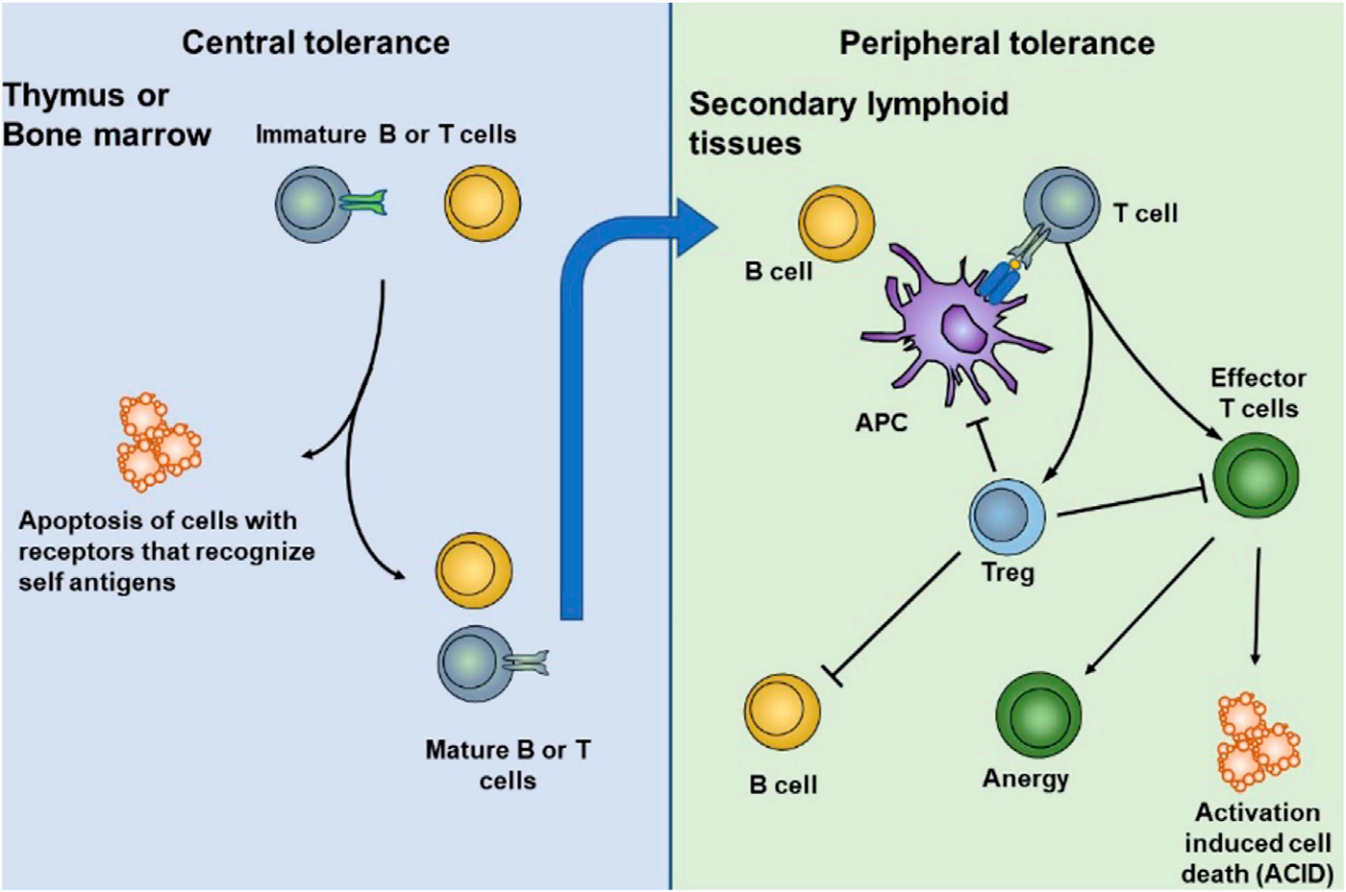
DCs play an essential role in central and peripheral immune tolerance. In central tolerance, most of the autoreactive T and B lymphocytes are cleared during the early stages of thymus and bone marrow development. Some self-reactive lymphocytes that escape central tolerance are cleared, anergized, deleted, or differentiated into normal T cells in peripheral tolerance. Reproduced with permission from (Yu et al., 2018).

Immune tolerance is primarily maintained through coordination between central and peripheral immune tolerance. In central tolerance, most of the autoreactive T and B lymphocytes are cleared during the early stages of thymus and bone marrow development. This process is also known as “negative selection.” Thymic DC plays an essential role in central tolerance, such as clone deletion, clone transfer, and clone diversion. Although central tolerance mechanisms are efficient, they cannot eliminate all self-reactive lymphocyte, partly because not all self-antigens are expressed at the primary site of lymphocyte development (Figure 2). Therefore, peripheral tolerance mechanisms exist, and these are crucial for controlling the tolerance of lymphocytes that first encounter their cognate self-antigens at the periphery (Xing and Hogquist, 2012).

Peripheral DCs are the inducers of immune responses and the crucial regulators of tolerance induction and maintenance. Many studies have focused on isolating and reprogramming dendritic cells (DCs) to generate tolerogenic DCs and maintain the immune tolerance environment. Tolerogenic DCs are mainly induced by various immunosuppressant drugs such as rapamycin (Macedo e al., 2012; Sukhbaatar et al., 2016), dexamethasone (Lee et al., 2017), and vitamin D (Xie et al., 2017; Kim et al., 2018) or inhibitory cytokines such as IL-10 (Boks et al., 2012; Kryczanowsky et al., 2016) and TGF-β (Rabinovich et al., 2007; Hasegawa and Matsumoto, 2018) to cultivate dendritic cells *in vitro* and then transfuse them back into the body to treat autoimmune diseases. Tolerogenic dendritic cells (Tol DCs) are characterized by reduced expression of costimulatory molecules and MHC class molecules, decreased ability to induce T-cell proliferation, and/or increased differentiation to regulatory T cells (Tregs) (Lee et al., 2017). Despite initial indications of therapeutic efficacy in some trials, the widespread treatment of Tol DCs remains challenging because the Tol DC induced *in vitro* is nonspecific, expensive, and maybe off-target after transfer *in vivo* (Cifuentes-Rius et al., 2021). Moreover, inducing disease, specifically immune suppression without impairing normal immune function, requires loading these DCs with disease-specific autoantigens (Stabler et al., 2019). Therefore, it is necessary to induce Tol DC to inhibit abnormal cytotoxicity and inflammatory responses in an antigen-specific manner, providing a precise approach for treating autoimmune diseases *in situ* (Gallucci et al., 1999).

Significantly due to the special tolerogenic environment of the liver, there are many research studies on the treatment of autoimmune diseases by targeting antigen-presenting cells in the liver. The liver is a well-known tolerogenic organ, which is constantly exposed to a mass of harmless gut-derived bacterial or commensal antigens from the gastrointestinal tract (Racanelli and Rehermann, 2006; Thomson and Knolle, 2010). The maintenance of hepatic tolerance is mediated by a series of liver-resident antigen-presenting cells, including dendritic cells, Kupffer cells (KCs.), and liver sinusoidal endothelial cells (LSECs) (Carambia et al., 2015; Doherty, 2016).

Many nanoparticles were enriched in the liver after administration *in vivo*. Some of them were internalized by KCs, which play a vital role in antigen presentation and tolerance induction (Horst et al., 2016). As the liver-resident macrophages, they phagocytose pathogens, dead-cell debris, and other alien invaders, such as nanoparticles at about 500 nm in size range. Heymann demonstrated that KCs induced hepatic tolerance protected mice from kidney inflammation in T cell-mediated glomerulonephritis, mainly by mediating T cell arrest and Treg expansion (Heymann et al., 2015). LSECs are special microvascular endothelial cells that are the second type of scavenger cells in the liver. LSECs mainly phagocytose particles at about 200 nm in size range by clathrin-mediated endocytosis (Sorensen et al., 2012), such as small particles and soluble macromolecules, which are mainly from circulation or processing by splenic cells (Thomson and Knolle, 2010; Carambia et al., 2015). LSECs can efficiently suppress inflammatory T cells response (Limmer et al., 2000; Klugewitz et al., 2002) and induce Tregs (Carambia et al., 2014). Carambia showed that targeting LSECs with polymer-coated MBP-coupled NPs administration injection protected mice from CD4^+^T cell-driven EAE by inducing Foxp3^+^ Treg cells (Carambia et al., 2015). This team then used SIINFEKL peptide-loaded nanoparticles, which are high selectively, internalized by LSECs *in vivo* and prevent CD8 T-cell driven autoimmune cholangitis (Carambia et al., 2021). Qi Liu et al. used biodegradable PLGA polymers to encapsulate OVA, targeting LSECs to induce antigen-specific immune tolerance in allergic airway disease. LSEC-targeting NPs dramatically suppress airway allergic inflammation by tissue infiltrating Tregs, promoting anti-inflammatory cytokines (Liu et al., 2019).

In general, nanomedicine offers a new way to overcome the above problems by loading a certain amount of pathogenic antigen onto DC *in vivo*, codelivery of some immunomodulatory substances that contribute to tolerance and performing DC reprogramming *in situ* (Cifuentes-Rius et al., 2021). This approach allows tolerogenic DC to be widely used in the clinic and easily applied to many different autoimmune diseases. However, nanoparticles also have many problems affecting their function as carriers.

## 3 Optimizing Nanoparticle’s Properties

**FIGURE 3:**
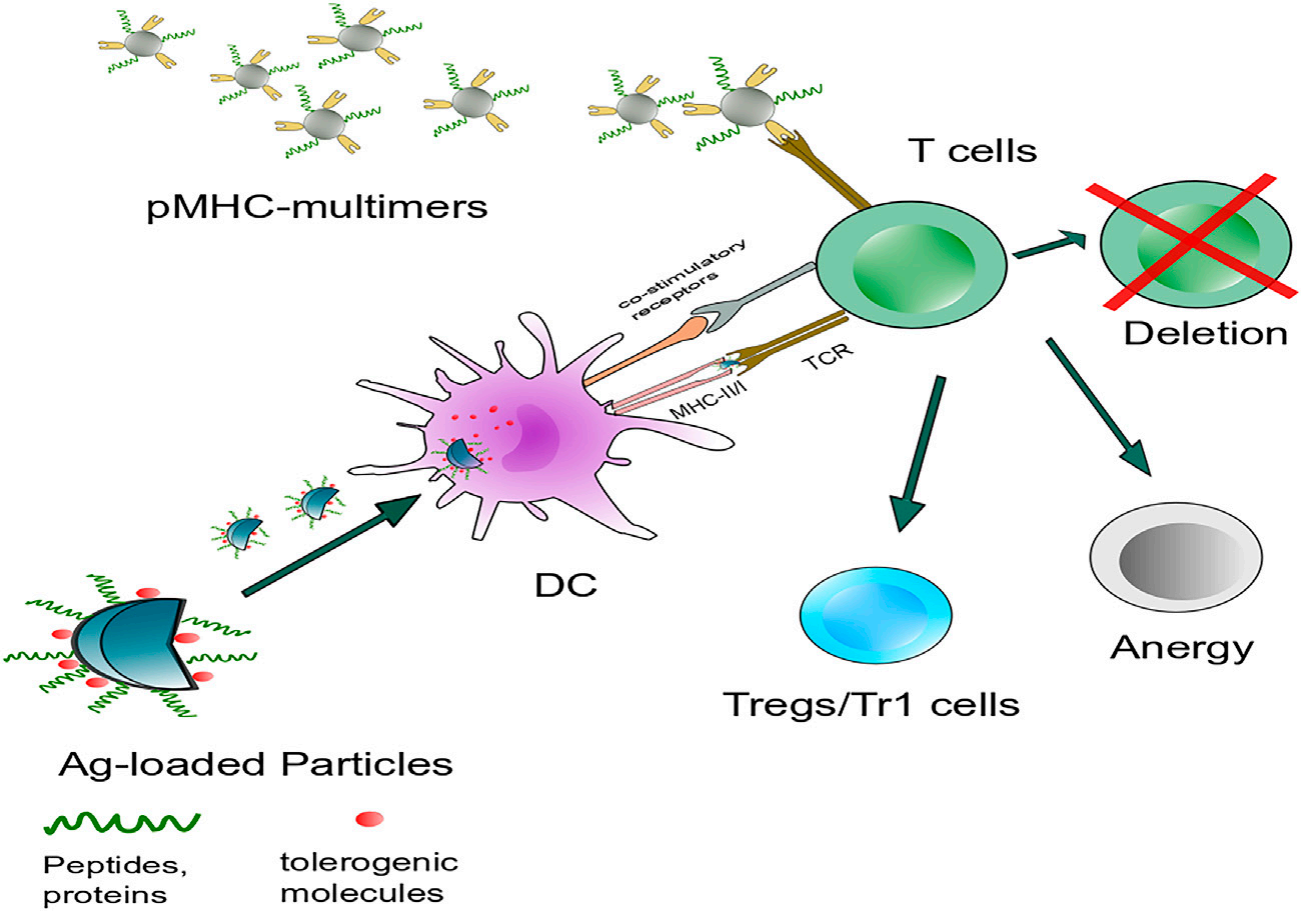
Intervention of antigen-specific autoimmunity is mainly concerned with two aspects: Targeting DCs or autoreactive lymphocytes to induce immune tolerance. Nanoparticles carry specific autoantigens with or without tolerogenic molecules that target antigen-presenting cells such as DCs *in vivo* and interfere with autoreactive T cells, including autoreactive T cell anergy and apoptosis and the induction of Tregs or Tr1 cells. Another strategy is systemic delivery of NPs. Coated with disease-relevant pMHC multimers targeting cognate autoantigen-experienced T-cell directly, leading to the formation and expansion of cognate TR1 cells.

Nanoparticles (NPs) have significant potential as a tolerance delivery vehicle with several benefits to autoimmune disease, allergy, and transplantation rejection immunotherapy. Some primary objectives should be designed to induce tolerance to a specific direction and avoid unnecessary immunosuppression (Liu et al., 2021). A certain amount of peptide is processed by DC, and presented as pMHC multimer to T cell, inducing lymphocyte activation. Interfere with one or multiple progress in lymphocyte reaction could induce apoptosis of autoactivated T cells or differentiation toward regulatory T cells (Yu et al., 2018). In this process, an ideal carrier: 1) should be able to protect the peptide cargo from the degradation of the internal environment *in vivo*, 2) can deliver cargo antigen to specific cells, such as DC (Lewis et al., 2019), macrophage (Montes-Cobos et al., 2017), 3) requires non-toxicity and biodegradability, and does not have apparent characteristics of inducing inflammatory activation. Moreover, NPs can decrease drug dosage and adverse reactions to organisms (Figure 3) (Urbanavicius et al., 2018; Cifuentes-Rius et al., 2021). Immune tolerance can be affected by the physicochemical characteristics of NPs, such as the size of the particles that can determine their internalization and biological distribution *in vivo*. The shape plays a role in the particle circulation time, biodistribution, and immune targeting (Thorp et al., 2020). The surface charge influences solubility, uptake by APCs, and DC maturation (Jazayeri et al., 2021; Lu et al., 2021).

### 3.1 Material Composition of Nanocarriers

At present, there are many biomaterials used for DC stimulation to suppress or activate immune responses. To achieve targeted delivery, nanoparticles are prepared based on design principles. The materials selected as vehicles should meet the main requirements, such as biocompatibility, non-toxic, easy to manipulate in size, and chemical properties (Urbanavicius et al., 2018). Some high immune stimulation materials, such as aluminum salt adjuvants, are unsuitable for inducing tolerance (Huang et al., 2020). Among all the materials used for nanoparticle design, synthetic polyester-based polymers, including polylactic acid (PLA) and poly (lactic-co-glycolic acid) (PLGA), are the most widely used materials for the preparation of nanoparticles. The advantages of these types of polymer materials for nanoparticles are their long shelf life and the ease of synthesis that allows for the encapsulation of various hydrophobic and hydrophilic compounds as well as proteins (Devulapally et al., 2016; Bee et al., 2018). Moreover, these polymers are biocompatible and biodegradable and cause slight immune reactions. Its metabolites are mainly carbon dioxide and water, therefore having low toxicity (Amini et al., 2017; Elmowafy et al., 2019). These PLGA degradation products are lactic acid and glycolic acid, which reduces the environmental pH value and induce the body to produce an immune tolerance environment (Allen et al., 2018; Bee et al., 2018). The underlying mechanism is related to the interference of the inflammatory signal by lactic acid. Lactic acid (LA) accumulates under inflammatory conditions and may influence a variety of signal pathways, such as NF-κB signal (Samuvel et al., 2009) and the PI3K/AKT pathway (Mendler et al., 2012). Peter considered that LA delayed the phosphorylation of protein kinase B (AKT) and the degradation of IκBα. Consistently, the LPS-induced genes delayed, and diminished NF-κB nuclear accumulation on monocytes and effector protein TNF-α, IL-23 which might contribute to immune suppress (Peter et al., 2015).

The liposome is another common drug carrier similar to the natural composition of cell membranes, which has a high degree of biocompatibility and lower entry barriers. Liposomes are amphiphilic lipid bilayer vesicles composed of phospholipids such as phosphatidylcholine, phosphatidylethanolamine, phosphatidylserine, and phosphatidylglycerol, and a stabilizer, such as cholesterol (Knudsen et al., 2015; Bulbake et al., 2017; Cong et al., 2020; Guo et al., 2021). Compared with polymeric or synthetic nanoparticles, liposomes have natural advantages as tolerogenic materials, partly because lipids are natural components of cell membranes and therefore are less likely to induce inflammatory reactions (Knudsen et al., 2015; Bulbake et al., 2017). Liposome-based platforms have been successfully employed as anti-tumor drug delivery in clinical applications, such as Doxil®, the first FDA-approved PEGylated liposome-based drug delivery (Dai et al., 2019). Many lipid nano-carries have been developed to treat anti-inflammatory (Serhan et al., 2008; Serhan and Petasis, 2011) or induce tolerogenic immune response (Basomba et al., 2002; Nielen et al., 2004; Nouri et al., 2015). Studies have shown that negatively charged lipids, (phosphatidylserine, PS) can induce tolerance *in vivo*. The mechanism may be that these particles can mimic the effects of apoptotic bodies, and inhibit DC maturation (Shi et al., 2007; Benne et al., 2016). Dongmei Shi et al. demonstrated that PS could induce tolerogenic DCs and suppressed CD4^+^T cell proliferation and IFN-γ production (Shi et al., 2007). Another study also found that PS-liposome phagocytosis resulted in phenotypic and functional changes in human DCs, which were accountable for tolerance induction (Rodriguez-Fernandez et al., 2018). Another mechanism by which liposomes induced tolerance was immunosuppression through the NF-κB signaling pathway (Chambers et al., 2018; Svajger and Rozman, 2020). Compared with free corticosteroids, the liposomal drug delivery system effectively inhibited arthritis, indicating promising therapeutic effects in autoimmune diseases (Meka et al., 2019). In some experimental arthritis models, liposomes-loaded with glucocorticoids and cytotoxic drugs showed a persistent anti-inflammatory effect, low dosage, and minor adverse reactions compared with free drug (A.S. Williams, 2001; Metselaar et al., 2003; Rauchhaus et al., 2009).

Polysaccharides are another frequently designed vaccine delivery system because of their biocompatibility, biodegradability, and low toxicity. Natural polysaccharides may be neutral (amyloglucan), or charged (hyaluronic acid and chitosan), and mainly are bio-adhesive to mucous membranes and the epithelium (Serrano-Sevilla et al., 2019). They are diverse in structure and size, often load various immunosuppressive drugs and antigens through electrostatic adsorption and selectively deliver immunosuppressive cargos to specific targets. Nanoparticle-mediated hyaluronic acid (HA) preferentially targets inflammatory tissues (for example, collagen-induced arthritis mice), which inhibits the expression of inflammatory cells such as macrophages, lymphocytes, and pro-inflammatory cytokines, ultimately reducing joint use inflammation and destruction (Shin et al., 2014), (Aguero et al., 2017; Barclay et al., 2019). Chitosan is a biopolymer derived from the deacetylation of chitin (Ding et al., 2014). Chitosan is extensively used to deliver nucleic acids because of its cationic nature through electrostatic interaction with the negatively-charged nucleic acid (Mikusova and Mikus, 2021).

Other nanomaterials, such as Au (Niikura et al., 2013; Sun et al., 2019) and pSi (Tieu et al., 2018; Kim B. et al., 2019), are also designed to target DCs and form immune tolerance because of their physical and chemical properties and surface chemical structures. However, nanomaterials themselves are only one parameter to be considered when designing nanomaterials. Nanoparticle size and shape also affect biological function and immune distribution.

### 3.2 Effect of Size and Shape on Nanoparticles

The size of nanoparticles mainly affects their circulation, internalization, and biological distribution *in vivo*. In terms of particle size alone, particles smaller than 5 nm were mainly cleared in the kidney (Choi et al., 2007), while particles larger than 1 µm would be internalized by macrophages after entering circulation before reaching target tissues and organs (Bachmann and Jennings, 2010). Generally speaking, according to hemodynamic studies, the smaller the particle, the longer it is in the circulation and the more it accumulates in the cells (Thorp et al., 2020). The long tissue residence time prolongs the particle’s action time and means higher toxicity to the internal cells (Lovric et al., 2005; Huang et al., 2011; Wozniak et al., 2017). Nanoparticles with diameter smaller than 10 nm exhibited toxicity secondary to inefficient cellular clearance and prolonged cellular accumulation *in vivo* (Huang et al., 2011). Although large-sized particles usually mean high drug load potential, there are many problems associated with large-sized particles, such as blocked small vessels and pulmonary embolism (>1 µm). Those problems make them rarely used to induce immune tolerance by intravenous injection (Decuzzi et al., 2010) or more easily cleared by phagocytic cells in the bloodstream before reaching the target organ (Thorp et al., 2020).

Since particles are internalized by antigen-presenting cells after entering the circulation, particle size also affects the type of internalization. Studies among a series of cell lines and different nanoparticles show the ideal size for nanoparticles uptake relevant to the cell type (Shang et al., 2014). For some non-phagocytic cells (such as the B16 cell line, etc.), particles smaller than 200 nm in diameter are most taken up by clathrin-mediated processes (Zuhorn et al., 2002), larger particles (200 nm–1 μm) enter cells preferentially along the pathway of caveolae-mediated endocytosis(Rejman et al., 2004; Gratton et al., 2008). After internalization, about particles of around 200 nm accumulate in late endosomal or lysosomal compartments, through the late intracellular endosomal receptors, engaging both adaptive and innate immune process, which is considered beneficial for immunomodulation (Gleeson, 2014).

While in professional antigen-presenting cells, the size of nanoparticle may be only one parameter besides charge, shape, and the route of administration, as nanomaterials ranging from under 5 nm to more than 1 μm could be internalized by DCs successfully (Bachmann and Jennings, 2010). In general, nanoparticles administered intravenously are mainly internalized by macrophages in the reticuloendothelial system (for example, liver, spleen) (Patel et al., 2010; Wang et al., 2012) or captured by DCs in the blood and peripheral organs (Jia et al., 2018). Therefore, the ideal size of particle-induced tolerance is related to the target location. The larger particles (about 500 nm) are more likely to attract macrophages from the liver and spleen to initiate phagocytosis. There was much researches focused on the peptide coupled-PLGA/PLG/PS nanoparticle platform, which targets MARCO^+^ APCs in the marginal zone (Getts et al., 2012; Hunter et al., 2014; Hlavaty et al., 2016; Pearson et al., 2019; Freitag et al., 2020). Getts et al. speculated that MARCO played an important role in cell uptake of peptide-linked particles, macrophage antigen presentation, and/or antigen transfer to local dendritic cells (Getts et al., 2012). In addition, MARCO showed the effects in inhibiting inflammatory responses through inhibiting DC migration (Arredouani et al., 2007).

Nanoparticles at about 200 nm in diameter often rely on nonspecific internalization pathways, such as pinocytosis, microtubules, and clathrin (Getts et al., 2015; Behzadi et al., 2017). These particles predominantly accumulated in the red pulp of spleen (Ernsting et al., 2013; Serra and Santamaria, 2015) and LSECs in liver (Carambia et al., 2015), which allowed them to preferentially interact with immune cells at that location and made them ideal for inducing tolerance. At about 100–200 nm, the particles tend to circulate in the bloodstream for several hours and then enter the local draining lymph nodes, where resident DC captures them in the lymph nodes and induces early T cell immune responses. Nanoparticles larger than 200 nm circulate longer circulation life and enter lymph nodes mainly through migrating DC (Manolova et al., 2008; Rincon-Restrepo et al., 2017) and/or remain at the injection site (Engman et al., 2015; Rincon-Restrepo et al., 2017; Lewis et al., 2019). Nanoparticles with larger particle sizes are more likely to be accumulated in barrier cells before entering lymphatic vessels, making them relatively less targeted (Thorp et al., 2020). Therefore, the conclusion on ideal diameters for inducing tolerance remains widely divergent in the different findings.

The nanoparticles could be prepared in a variety of shapes. The biodistribution, cytotoxicity, circulation time, and immunogenicity of nanoparticles can be affected by their morphologies (Gratton et al., 2008). When designing nanoparticles, shape is often taken into account along with size (Firestein, 2003). Spherical nanoparticles have a lower tendency to marginate in the bloodstream and a longer circulation time than non-spherical nanoparticles, such as cubic, rod, and needle nanoparticles (Decuzzi et al., 2010). Spherical nanoparticles tend to be distributed homogeneously in most tissues and organs, especially preferential accumulation in liver, spleen, and lung (Gratton et al., 2008). The non-spherical nanoparticles usually have a higher surface area (cylinders, rods), which makes them more likely to be internalized by phagocytes, such as liver macrophages and circulating phagocytes (Gratton et al., 2008; Decuzzi et al., 2010; Huang et al., 2011). While the nonspherical nanoparticles tend to be more rigid, and are more likely to damage membrane during cell uptake, they may also cause further activation of the inflammasome in immune cells (Zhao et al., 2013; Wozniak et al., 2017).

### 3.3 Surface Properties of Nanocarrier

#### 3.3.1 Charge of the Particles

The nanoparticle charge is another critical character parameter that affects particle internalization and subsequent immune response. Generally, high charge (>30 mV), whether positive or negative, are generally more stable because of electrostatic repulsion (Yan et al., 2008; Hunter et al., 2014; Sun et al., 2014; Hlavaty et al., 2016; Saito et al., 2019a; Saito et al., 2019b). While charge is a dynamic physicochemical parameter, in biological microenvironment, proteins can adsorb to surface of particles, forming protein coronas which can lead to aggregation, macrophage uptake, and rapid clearance (Cifuentes-Rius et al., 2013). In general, cationic nanoparticles are more easily internalized by cells (Fytianos et al., 2015). Compared with anionic particles, cationic nanoparticles appear to be internalized rapidly by interacting with negatively charged cell membranes (He et al., 2010) or *via* the clathrin-mediated pathway (Harush-Frenkel et al., 2007) and are more suitable for inducing inflammatory responses (Yan et al., 2008; Saito et al., 2019b). Furthermore, positively particles tend to be taken up by DCs, which stimulates Th1 immune response consequently (Koppolu and Zaharoff, 2013; Fromen et al., 2015). Antigen encapsulated cationic NPs could induce elevated IgG2a titers and IFN-γ secretion by DCs after mucosal or intradermal vaccination, resulting in Th1 immune responses (Gursel et al., 2001; Bal et al., 2011; Getts et al., 2015).

On the other hand, negatively charged NPs show an inferior rate of internalization, and rarely through the clathrin-mediated pathway (Harush-Frenkel et al., 2007). Some studies show negatively charged particles have the effect of ameliorating inflammation in autoimmune diseases and chronic injury (Allen et al., 2018; Saito et al., 2019b). The underlying mechanism is that the particles were intravenously injected, targeting inflammatory cells in circulation directly, disturbing their migration to CNS (Fromen et al., 2017; Saito et al., 2019b) or phagocytosed by monocytes and neutrophils, inducing apoptosis, then sequestered in the spleen or liver for elimination, delayed infiltration into the CNS. What is more, it has been proved that nanoparticles with a charge below −30 mV have anti-inflammatory efficacy (Hunter et al., 2014; Hlavaty et al., 2016; Saito et al., 2019a). Some studies have shown peptide coupled nanoparticles (500 nm range) with negatively charge ranging from −40 to −70 mV could target phagocytes cells expressed MARCO receptors in the spleen or liver in an opsonin-independent manner, inducing antigen-specific tolerance (Getts et al., 2012; Hunter et al., 2014; Hlavaty et al., 2016; Pearson et al., 2019; Freitag et al., 2020).

However, the rate of internalization of a particle is not necessarily related to its ultimate potency. Studies on intestinal epithelial cells have found that cationic nanoparticles internalize rapidly, but their vesicular trans-monolayer transport is slow. In contrast, anionic nanoparticles have the opposite properties, which make them more efficient in cortical transport (Bannunah et al., 2014). In summary, surface charge influences particle internalization, transmembrane transport, and biological functions. However, it is only one parameter of surface properties in particles. Further surface modification is necessary to control the complex interactions between nanoparticles and cells.

#### 3.3.2 Surface Modification of Nanoparticles

Sometimes, it is necessary to modify the surface to prolong their circulation time, delivering to specific targets (Fan et al., 2020) or designing for desired therapeutic effects (Rieger et al., 2009). The clearance of nanoparticles by the reticuloendothelial system (RES) is the main obstacle to the delivery efficiency of nanocarriers. Many methods have been adopted to increase the half-life of nanoparticles. A commonly used means is coating particles with polymeric ethylene glycol (PEG). PEG-modified nanoparticles can increase the hydrophilic protective layer around the nanoparticles making it difficult to be adsorbed or collected by hemoglobin, thus prolonging the circulation time of particles in the blood (Gref et al., 2000; Casals et al., 2010). Previous studies have suggested that liposome particles with a slightly negative charge, modified with PEG (10%; 5 kDa), can prolong the circulation time *in vivo* and have better joint targeting, improving the therapeutic effect of RA (Hirai et al., 2007; Meka et al., 2018). PEO (polyethylene oxide) is the low molecular weight derivative of PEG, which forms a “mushroom-brush” or “brush” configuration on particles, avoiding clearance by scavenger cells. Unlike PEG, PEO may change activation from the C1q-dependent complement pathway to the lectin pathway. Meantime, PEO can also reduce the level of complement activation products (Hamad et al., 2010). When NPs are administrated by vein, some plasma protein (such as immunoglobulin G, complement factors, and fibrinogen), rapidly bind to NPs to form a protein corona, inducing macrophage recognition and phagocytosis (Gao and He, 2014; Shen et al., 2018) which is called particle opsonization. By contrast, replacing these proteins with to some serum albumins or lipoproteins could reduce the internalization and prolong circulation times. Another strategy to minimize recognition and phagocytosis is codelivery of a “self-marker,” such as CD47 molecules, with the NPs to prevent endocytosis. CD47 may interact with SIRPα expressed on phagocytes, and suppress endocytosis (Pei et al., 2018).

#### 3.3.3 Codelivery of Tolerant Payloads

Targeting DC to induce immune tolerance has been studied widely, including immune tolerance by mimicking apoptotic cell death (Turley and Miller, 2007; Luo et al., 2008; Kontos et al., 2013; Pozsgay et al., 2016; Clough et al., 2020), allowing antigens to utilize the immune tolerance environment of the body (Carambia et al., 2015; Park et al., 2016; Liu et al., 2019), or inducing tolerance by simultaneous coupling of some tolerant drugs or small molecules targeting DC (Yu et al., 2018). The mainly co-delivered tolerance cargo includes glucocorticoids, nucleic acids, and small molecule immunosuppressants.

Glucocorticoids are the most commonly used immunosuppressive drugs in the clinic, and their main pharmacological action is to dissolve active immune cells and block cell differentiation. They are characterized by non-specificity and are broad immunosuppressants. Some studies used PLGA-loaded glucocorticoids to treat multiple sclerosis, autoreactive arthritis, and ulcerative colitis, both of which achieved ideal target therapeutic effects, durable anti-inflammatory effects, and lower adverse reactions, such as metabolic syndrome disorder, hyperglycemia, hyperlipidemia, and hypertension (Nakase et al., 2003; Higaki et al., 2005; Montes-Cobos et al., 2017; Kim S.-H. et al., 2019).

Gene therapy is a great potential treatment for autoimmune disease; however, few clinically available options are available. There are several reasons for limiting the clinical use of nucleic acids, such as high molecular weight, instability in natural environments, enzymatic degradation, and inability to transport across the cell membrane (Lostalé-Seijo and Montenegro, 2018). A nanoparticle can protect siRNA (Guo et al., 2021), mRNA, microRNA, and plasmid DNA from a series of barriers. Currently, the most utilized nucleic acid nanocarrier delivery modules are cationic lipids and synthetic polymers. In addition, a chitosan nanoparticle loaded with Lingo-1 siRNA (a protein suppressing myelination and axonal regeneration) in the mouse model of demyelination showed neuroprotection and remyelination effects (Youssef et al., 2019). A PLGA microsphere codelivery peptide and an antisense oligonucleotide of costimulatory molecules can reverse the hyperglycemia in type 1 diabetic mice (Engman et al., 2015). Similar research used short interfering RNA (siRNA) to explicitly generate tolerogenic DCs by knockdown of CD40, CD80, and CD86 at the same time after injection the arthritogenic antigen collagen II [setting up the collagen-induced arthritis (CIA) model], which could effectively suppress the onset of collagen-induced arthritis. The results support using siRNA to generate tailor-made tolerogenic vaccines for treating autoimmunity (Zheng et al., 2010).

Small molecule compounds have advantages in pharmaceutical technology, stability, and safety compared with nucleic acids and some protein drugs. Small molecule immunomodulators, including vitamin D3 (Agmon-Levin et al., 2013; Keijzer et al., 2013), mycophenolic acid (Look et al., 2013), and rapamycin (Stabler et al., 2019), have been shown to effectively induce tolerance through various mechanisms, including the induction of Tregs or by altering the profile of pathogenic immunity. Codelivery of rapamycin, a natural macrolide compound that acts as an allosteric inhibitor of the mammalian target of rapamycin (mTOR) pathway, has been shown to have tolerogenic properties *in vitro* and *in vivo* (Maldonado et al., 2015; Tostanoski et al., 2016; Zhang et al., 2016). Some research also showed that rapamycin-carrying nanocarriers have potent immunosuppressive activity to inhibit T cell proliferation, and the possible mechanism of rapamycin-carrying nanocarriers is the inhibition of anti-drug antibodies. Takashi et al. evaluated a synthetic, biodegradable, nanoparticle codelivery disease-related peptide, SVP-rapamycin, to induce durable immune tolerance *in vivo* by treating animals in multiple autoimmune disease models such as experimental autoimmune encephalomyelitis (EAE) and rheumatoid arthritis (RA) in different species such as mice, rats, and monkeys. The results demonstrate the ability of SVP-rapamycin co-administered with antigen to induce tolerogenic DCs *in vivo* that can promote antigen-specific Tregs (Kishimoto et al., 2016). Similarly, SJL mice treated with nanoparticles containing rapamycin and pathogenic peptides protected the mice from developing EAE and induced antigen-specific tolerance (LaMothe et al., 2018). Coadministration of protein/pathogenic peptide antigen and the immunosuppressant rapamycin can induce durable and specific resistance to mounting immune responses toward the antigen in animal models of hemophilia A and relapsing-remitting model of experimental autoimmune encephalomyelitis (Maldonado et al., 2015). Sialic acid (SA), a 9-carbon carboxylated monosaccharide, is mainly located on the surface of cell membranes. As the binding ligand of E-selectin receptors, SA can improve transportation and accumulation of micelles in inflammatory cells. Methotrexate loaded SA-dextran-octadecanoic acid micelle (SA-Dex-OA/MTX) to inhibit the inflammatory response, reduce the side effects of methotrexate and enhance the bone repair activities in the treatment of RA (Xu et al., 2018). The aryl hydrocarbon receptor (AhR) is a ligand-activated transcription factor, which is another candidate to target DC because of its modulation in the differentiation of regulatory T cell subsets (Quintana et al., 2010; Takenaka et al., 2019). Nanoliposome-loaded AhR ligand (ITE) and disease-specific peptide antigens were used to induce antigen-specific tolerance and suppress EAE in mouse models of multiple sclerosis (Yeste et al., 2012; Rothhammer et al., 2017).

In the course of the onset or progression of autoimmune diseases, autoreactive T-lymphocytes produce inflammatory cytokines, resulting in inflammatory reactions or organ and tissue damage. Some therapeutic strategies have been proposed to block inflammatory cytokines or increase anti-inflammatory cytokines. Cytokines [such as IL-10 (Cappellano et al., 2014) and TGF-β (Casey et al., 2018)] are often delivered to antigen-presenting cells together with antigens to induce antigen-specific immune tolerance. However, there are still some problems associated with cytokines used as therapies. *In vivo*, a variety of cytokines play a synergistic role, so the effect of the cytokine is often context-dependent. For example, although IL-2 and TGF-β induce Tregs, IL-17 and TGF-β induce pro-inflammatory Th17 cells (Horwitz et al., 2019).

## 4 Summary and Prospects

Autoimmune diseases are chronic inflammatory diseases involving multiple cells and systems, and traditional systemic immunosuppression cannot meet the requirements of precise treatment. Nanoparticle-mediated delivery-induced tolerance *in vivo* is a promising strategy in autoimmune disease or transplantation. The ability of particles to efficiently deliver antigens and immunomodulators, mainly targeting antigen-presenting cells and lymphocytes, can increase the ability to induce specific tolerance. Targeted delivery of protected antigens directly to immune cells ensures efficient, safe, and nonspecific damage. In addition, there are many strategies to optimize nanoparticles for a better fit for immune tolerance therapy, such as controlling the localization, dose, and kinetics of tolerogenic particles. These studies have resulted in many remarkable results. The first-in-man, open-label, single-center clinical trial in RR and SP (secondary progressive) MS patients (ETIMS trial) involved autologous peripheral blood mononuclear cells chemically coupled with seven myelin peptides in the presence of the chemical cross-linker 1-ethyl-3-(3-dimethylaminopropyl)-carbodiimide (EDC) in MS patients, establishing the feasibility and indicating good tolerability and safety of this therapeutic approach(Lutterotti et al., 2013).

Great efforts have been made to develop nanotechnology-induced tolerance during the last decades. It has been more than 30 years since the first nanotechnology vaccination against tetanus toxoid (Birrenbach and Speiser, 1976). Although it has successful experience in human clinical practice (Table 1), it is still difficult to translate this therapy into the clinic, as several factors limit its clinical transformation. First, there are noticeable species differences between animals and humans in genetic inheritance and living environment, so the guidance of animal models for actual diseases is limited. Second, animal models of diseases often adopt a single antigen to simulate disease models. However, most human autoimmune diseases are characterized by antigen heterogeneity and epitope expansion or variation, which results in animal models being unable to accurately simulate clinical disease models. This occurred with the first nano-vaccine that co-delivered seven pathogenic peptides at one time and many more autoimmune disease epitopes that were not clear. In addition, animal experiments used prevention to interfere with the disease process, while human diseases tend to develop insidiously and delay for several years. Patients often were presented with obvious clinical symptoms or sequelae during observation, indicating that their disease is in the progression or sequelae stage, making the treatment more challenging (Pearson et al., 2017). In the long term, antigen-specific immune tolerance based on nanoparticles may be a good focus area for research on autoimmune diseases, but much more effort is still required.

**TABLE 1:** Some clinical trials on nanoparticle therapies for tolerance induction.

Disease	Properties of nanoparticles	Administration method	Codelivery antigen/drug	Phase	Reference
Type 1 diabetes	Gold	Intradermally	Proinsulin-derived peptide (C19-A3 GNP)	Phase I	https://clinicaltrials.gov/ct2/show/NCT02837094
Plaque psoriasis	Uncoated nanoparticle paclitaxel ointment (SOR007)	External use	Paclitaxel	Phase I	https://clinicaltrials.gov/ct2/show/NCT03004339
RA	Liposomes	Subcutaneous injection	Prednisolone	Phase II	https://clinicaltrials.gov/ct2/show/NCT00241982
RA	PEGylated nanomolecules (Pegsunercept)	Subcutaneous injection	TNFα inhibitor	Phase II	https://clinicaltrials.gov/ct2/show/NCT00111423
RA	PEGylated nanomolecules (Pegsunercept)	Subcutaneous injection	TNFα inhibitor	Phase II	https://clinicaltrials.gov/ct2/show/NCT00037700
RA	Liposome	Subcutaneous injection	NF-kB inhibitor 1,25 hydroxyvitamin D3(calcitriol)	Phase I	2019 ACR/ARP Annual Meeting Archives - ACR Meeting Abstracts (acrabstracts.org)
Coeliac disease	PLG [poly(lactide-co-glycolide)] nanoparticles	Splenic marginal zone macrophages and liver phagocytic cells via scavenger receptors (MARCO)	Gliadin	Phase I	Freitag et al., (2020)
